# Reduced Educational Outcomes Persist into Adolescence Following Mild Iodine Deficiency in Utero, Despite Adequacy in Childhood: 15-Year Follow-Up of the Gestational Iodine Cohort Investigating Auditory Processing Speed and Working Memory

**DOI:** 10.3390/nu9121354

**Published:** 2017-12-13

**Authors:** Kristen L. Hynes, Petr Otahal, John R. Burgess, Wendy H. Oddy, Ian Hay

**Affiliations:** 1 Menzies Institute for Medical Research, University of Tasmania, Private Bag 23, Hobart, TAS 7001, Australia; Petr.Otahal@utas.edu.au (P.O.); Wendy.Oddy@utas.edu.au (W.H.O.); 2 Department of Endocrinology, Royal Hobart Hospital, 48 Liverpool Street, Hobart, TAS 7000, Australia; J.Burgess@utas.edu.au; 3 School of Medicine, University of Tasmania, 17 Liverpool Street, Hobart, TAS 7000, Australia; 4 Faculty of Education, University of Tasmania, Locked Bag 1307, Launceston, TAS 7250, Australia; I.Hay@utas.edu.au

**Keywords:** iodine nutrition, iodine deficiency, gestation, educational outcomes, literacy, children, adolescence, working memory, auditory processing speed

## Abstract

There is increasing evidence that even mild gestational iodine deficiency (GID) results in adverse neurocognitive impacts on offspring. It’s unclear, however, if these persist long-term and whether they can be ameliorated by iodine sufficiency in childhood. We followed a unique cohort (Gestational Iodine Cohort, *n* = 266) where gestation occurred during a period of mild population iodine deficiency, with children subsequently growing-up in an iodine replete environment. We investigated whether associations between mild GID and reductions in literacy outcomes, observed at age 9-years, persisted into adolescence. Comparisons were made between offspring of mothers with gestational urinary iodine concentrations (UICs) ≥ 150 μg/L and < 150 μg/L. Educational outcomes were measured using Australian National Assessment Program—Literacy and Numeracy (NAPLAN) tests. Children whose mothers had UICs < 150 μg/L exhibited persistent reductions in spelling from Year 3 (10%, −41.4 points (95% Confidence Interval −65.1 to −17.6, *p* = 0.001)) to Year 9 (5.6%, −31.6 (−57.0 to −6.2, *p* = 0.015)) compared to children whose mothers had UICs ≥ 150 μg/L. Associations remained after adjustment for biological factors, socioeconomic status and adolescent UIC. Results support the hypothesis that mild GID may impact working memory and auditory processing speed. The findings have important public health implications for management of iodine nutrition in pregnancy.

## 1. Introduction

Insufficient iodine during gestation, particularly in the first trimester, is a major cause of preventable neurological damage [1,2]. While the deleterious impacts of severe iodine deficiency (ID) are well established, ID occurs along a continuum and there is increasing evidence that even mild ID can have subtle but measurable impacts on the offspring. In 2013, two landmark observational studies highlighted the consequences of mild gestational iodine deficiency (GID). In the UK, researchers reported reductions in intelligence quotient (IQ) measures, including verbal IQ, reading accuracy and reading comprehension, at age 8-years, in children of mothers classified with mild-to-moderate GID (iodine-creatinine ratio < 150 μg/g) [3]. Our team, similarly reported that 9-year-old Australian children had reduced educational performance in literacy, but not numeracy, assessments if their mothers had urinary iodine concentrations (UICs) < 150 μg/L during pregnancy compared to children whose mothers had UICs ≥ 150 μg/L [4].

Subsequent studies support these findings: Moleti et al. [5] described defective cognitive function, particularly verbal abilities, in Italian children (aged 6–12 years) whose mothers had mild GID and; a study of 3-year-old Norwegian children reported language delay in those whose maternal gestational iodine intake was below the Estimated Average Requirement of 160 μg/day [6]. Further support is also found in a study investigating the impacts of perchlorate, a known inhibitor of thyroidal iodine uptake, on cognitive development. Talyor et al. [7] reported verbal, but not performance, IQ was lower in the offspring (aged 3-years) of mothers with sub-optimal thyroid function and higher perchlorate levels in the first trimester.

While the evidence indicates that even mild GID can impact adversely on neurocognitive outcomes in childhood, two things remain unclear. Are the deleterious impacts of in utero iodine insufficiency long-lasting and can they be ameliorated by adequate iodine nutrition in childhood? We are uniquely placed to examine these questions, having a cohort whose gestation occurred during a documented period of mild ID (median UICs 72 to 75 μg/L between 1998 and 2000) [8] in the Tasmanian population, with the children subsequently growing up in an iodine replete environment (median UICs 105 to 130 μg/L, between 2003 and 2016) [9,10], following population iodine prophylaxis via fortification of bread with iodized salt in late 2001 [11,12]. In this follow-up of the Gestational Iodine Cohort we investigate whether our previously observed association between mild GID and reduced educational outcomes in literacy, at age 9-years [4], persists into adolescence. We discuss the role of iodine nutrition in childhood and explore possible mechanisms for the association including, deficits in hearing, central auditory processing, auditory processing speed and working memory.

## 2. Materials and Methods

The Gestational Iodine Cohort was established in 1999–2000 and the methods have been published previously [4]. In brief, women attending antenatal clinics at the Royal Hobart Hospital (Australia) provided between one and three random spot urine samples for iodine analysis. UIC was determined at the Institute of Clinical Pathology and Medical Research (IOS/IEC 17025 accreditation) using the modified Sandell-Kolthoff reaction [13] and is reported as micrograms of iodine per liter of urine (μg/L). In the absence of a more appropriate individual classification of iodine nutrition status, World Health Organization (WHO) population median UIC cut-points of ≥150 μg/L and <150 μg/L were used to classify the mothers into two groups [14] and for the purposes of this study the terms “sufficient” and “deficient”, respectively, are used to describe the iodine status of the groups. The UIC cut-point of 150 μg/L for iodine sufficiency during pregnancy, which is higher than the general population cut-point of 100 μg/L, reflects the increased requirement for maternal iodine during fetal development.

### 2.1. National Assessment Program—Literacy and Numeracy (NAPLAN) Study

Data linkage techniques were used to link offspring with NAPLAN outcomes assessed between 2008 and 2017 in Years 3, 5, 7 and 9 when the offspring were aged 8–9, 10–11, 12–13 and 14–15 years, respectively. NAPLAN tests are standardized criteria-referenced measures of individual student performance in literacy (reading, writing, and language conventions (spelling, and grammar and punctuation)) and numeracy. Testing is conducted annually by the Australian Federal Government in all schools. The Tasmanian State Government Department of Education (DoE) maintains a NAPLAN database of Tasmanian children and facilitated linkage to the gestational data. The DoE also provided socio-economic status (SES) measures (maternal and paternal level of education, maternal and paternal occupation, and indigenous status), which were collected when the children started school.

### 2.2. Comprehensive Evaluation of Language Fundamentals (CELF-4) and Central Auditory Processing Disorder (CAPD) Study

In 2013–2015, a preliminary investigation of possible mechanisms to explain the association between mild GID and reduced literacy outcomes in childhood was conducted. Funding was sufficient to test a subset of the Gestational Iodine Cohort; as such, only offspring born in 2000 were invited to participate. The Comprehensive Evaluation of Language Fundamentals (Fourth edition—Australian standardized edition) (CELF-4) [15] was used to determine whether GID was associated with specific delays in language development that may be related to deficits in NAPLAN literacy outcomes. A Central Auditory Processing Disorder (CAPD) assessment (developed by the National Acoustic Laboratories) was used to determine whether mild GID was associated with deficits in hearing and/or a central auditory processing disorder.

The CELF-4 is designed for diagnosing language disorders in 5–21 year-olds. Details are contained in the CELF-4 examiner’s manual [15] and Paslawski’s review [16]. All available age-appropriate subtests were administered and indexes calculated; where applicable results were age-scaled using Australian norm-referenced scores [15].

The CAPD protocol consists of four standardized tests used in combination to identify CAPD:

1. Hearing acuity assessed using unmasked air-conduction pure-tone audiometry. Normal hearing classified as a threshold of ≤20 decibels.
2. The Listening in Spatialized Noise-Sentences Test (LiSN-S) measured children’s ability to use spatial cues to help them understand speech in background noise (as experienced in classrooms) to assess their binaural processing skills. Protocol details have been published previously [17].
3. Two tests of auditory memory, Number Memory Forwards (NMF) and Number Memory Reversed (NMR) from the Test of Auditory Processing Skills-Third Edition (TAPS-3) protocol [18] were administered.
4. A Dichotic Digits Test (DDT) was used to assess binaural integration, defined as the ability to process information presented to both ears simultaneously when the information presented to each ear is different. The percentage of correctly repeated digits for each ear and the Right Ear Advantage (REA) were calculated. Handedness of participants was recorded.

The CELF-4 and CAPD were administered between 8:30 a.m. and 12:00 p.m. to reduce impacts of fatigue that may decrease attention span and effect assessment outcomes. Participants also provided a spot-morning urine sample, with UIC determined using the same laboratory and assay as the maternal samples.

### 2.3. Statistical Techniques

Means and standard deviations (SD) are presented for continuous measures and percentages for categorical measures. UIC was skewed; thus, median and interquartile range (IQR) are presented. Chi-squared tests were used to show group differences for categorical data and Student’s *t*-tests and Mann-Whitney *U*-tests were used for continuous data, where appropriate. As a preliminary step, univariable regression models of NAPLAN outcomes in Year 3, 5, 7 and 9 with gestational UIC as a continuous variable were examined. All subsequent models included UIC as a dichotomous variable, using the ≥150 vs. <150 μg/L cut-point detailed above. Mixed-effects regression models with a random intercept for individuals were used to analyze the repeated NAPLAN outcomes from Years 3 through 9 with gestational iodine status (UIC ≥ 150/< 150 μg/L). In these models, year was included as a categorical covariate to fit the non-linear pattern over time; further, an interaction with gestational iodine status and year was included to determine if differences between GID groups changed over time. Models are presented unadjusted, adjusted for biological covariates (gestational age at UI collection, maternal age, gestational length, birth weight and sex), with additional adjustment for the socio-economic covariate maternal education and finally for adolescent UIC. Model covariates were included in models for reasons of clinical importance and potential confounding. All mixed model analyses had some level of missing data in covariates and outcomes (5–30%), this was addressed with multiple imputation using chained equations (MICE) procedure combining 20 imputed datasets under Rubin’s Rules. All NAPLAN models are presented with imputed data.

Linear regression models were used in the CELF-4 and CAPD analyses. The level of missing data (non-response) was extreme (>80%), this combined with the lack of good imputation variables for predicting language and auditory outcomes resulted in unstable MICE models with very large standard errors. Inverse probability weighting was also not possible due to a very low number of variables with complete data for individuals. Therefore, only the linear regression analyses are presented.

Stata/IC12.1 was used for statistical analysis. Statistical significance was defined at *p* < 0.05.

Ethics approval granted by the Tasmanian Health and Medical Human Research Ethics Committee (Ref. Nos. H11592 and H13327). Informed consent of participants was obtained.

## 3. Results

### 3.1. NAPLAN Study

From the original Gestational Iodine Cohort, 266 singleton offspring were successfully linked to NAPLAN data. Examination of gestational measures (Table 1) revealed no meaningful differences between those followed-up and those not.

Four-hundred and forty-nine maternal urine samples were collected (between October 1999 and December 2001); 132 women provided one sample, 85, two samples and 49, three samples. The overall median UIC (using the mean UI for each pregnancy) was 83.2 μg/L (IQR: 46.0–180.0 μg/L), indicating mild ID. Mean gestational age at UI collection was 23.7 (SD 9.7) weeks (range: 6–41 weeks). Using the WHO population-based criteria cut-point for iodine nutrition during pregnancy [14] 69.2% (184/266) of the women had UICs < 150 μg/L. Table 2 shows there were no meaningful differences between the sufficient and deficient UIC groups for any gestational, birth, or SES measures.

**Figure 1:**
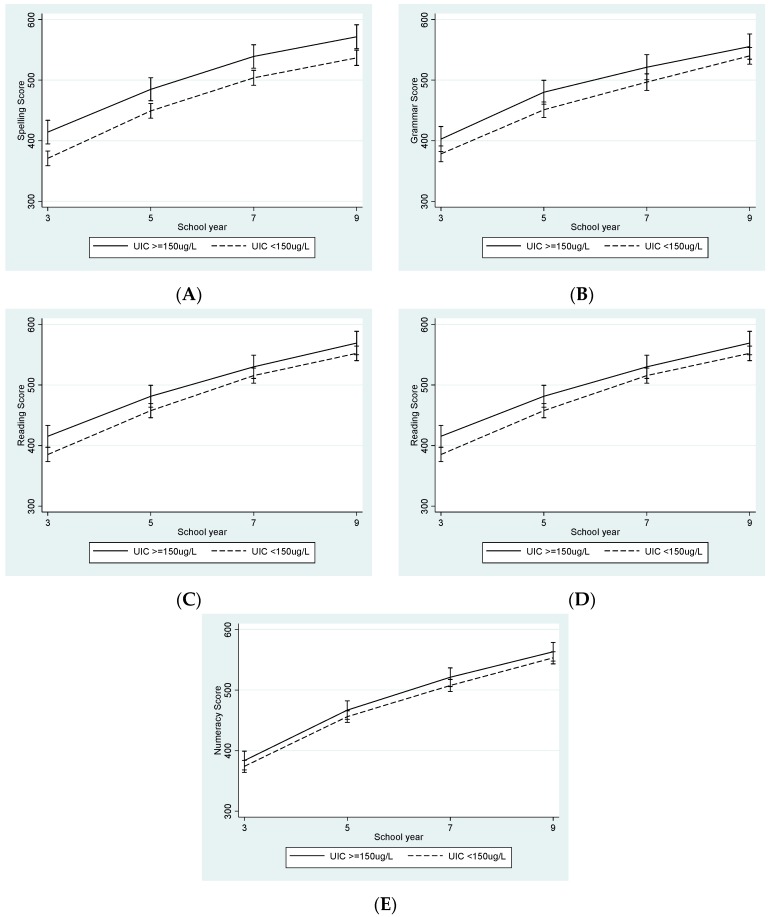
National Assessment Program–Literacy and Numeracy (NAPLAN) Scores for (**A**) Spelling, (**B**) Grammar, (**C**) Reading, (**D**) Writing and (**E**) Numeracy from School Year 3 to Year 9 by maternal urinary iodine concentration (UIC), using data from the final mixed-effects multiple imputation using chained equations (MICE) models adjusted for biological factors, socio-economic status (SES) and adolescent UIC (*n* = 266).

Table 2 also shows the NAPLAN scores for each testing year by UIC grouping, with the sufficient group having higher scores than the deficient group for all tests at each time point. Univariable examination of UIC as a continuous variable showed associations with all NAPLAN outcomes for the majority of the testing years (Table 3). NAPLAN MICE regression models are also shown in Table 3, with Figure 1 showing the differences over time for the UIC groups using outcomes from the fully-adjusted models.

For spelling, the differences at Year 3 between the deficient and sufficient groups, decreased in magnitude but remained meaningfully different as schooling progressed. Within each testing year, the differences remained unchanged in magnitude after adjustment for gestational factors and following further adjustment for possible confounding by maternal education and adolescent UIC. Using the fully-adjusted model, spelling outcomes in Years 3, 5, 7 and 9 were 10.0%, 6.6%, 6.1% and 5.6% lower in the iodine deficient group, respectively. For grammar and reading, differences at Year 3 continued into Year 5 but decreased by Year 9. An initial 6.5% difference in Grammar reduced to 2.8% by Year 9 and a 7.1% difference in reading reduced to 2.5% by Year 7, remaining steady in Year 9. NAPLAN writing and numeracy outcomes did not show large differences at any time-point in any of the models, with the exception of Year 5 writing. Widening of the gap (seen as the steeper trajectory of the iodine sufficient group between Year 3 and 5 in Figure 1) coincided with a switch from a narrative to a persuasive writing task for that particular year.

### 3.2. CELF-4 and CAPD Study

Sixty-six of the 75 original Gestational Iodine Cohort born in 2000 were traced. Of these 20 were either ineligible (moved interstate) or refused consent. A total of 46 offspring (now aged 13–14 years) participated in the CELF-4 and CAPD assessments. One was later excluded, being the only participant with earlier diagnosed learning difficulties, low birth-weight (<2500 g) and pre-term birth (<37 weeks).

Table 1 shows a number of differences in the characteristics of participants and the remaining cohort: participants had older mothers; none were classified as preterm or had low birth weight; maternal UIC was measured earlier in pregnancy; parental education and occupation were indicative of higher SES and; NAPLAN scores were higher. Among the participants, however, no differences in gestational or SES measures were found between the sufficient and deficient UIC groups (Table 2). The difference between the NAPLAN scores of the UIC groups in the CELF-4/CAPD participants was larger than the differences in NAPLAN for the whole cohort (Table 2).

Specific language disorders were not evident in any participants, with both UIC groups within age appropriate norms for language development. All CELF-4 measures (Table 4) were lower for offspring of iodine deficient mothers. The Formulated Sentence (FS) sub-test and the Expressive Language Index (ELI) (which includes FS), showed the greatest differences between groups. Regression modelling, using just the 45 participants, showed consistently reduced performance in the deficient group for the CELF-4 outcomes in unadjusted and adjusted models (Table 4), with the FS sub-test and the ELI showing the greatest differences.

**Figure 2:**
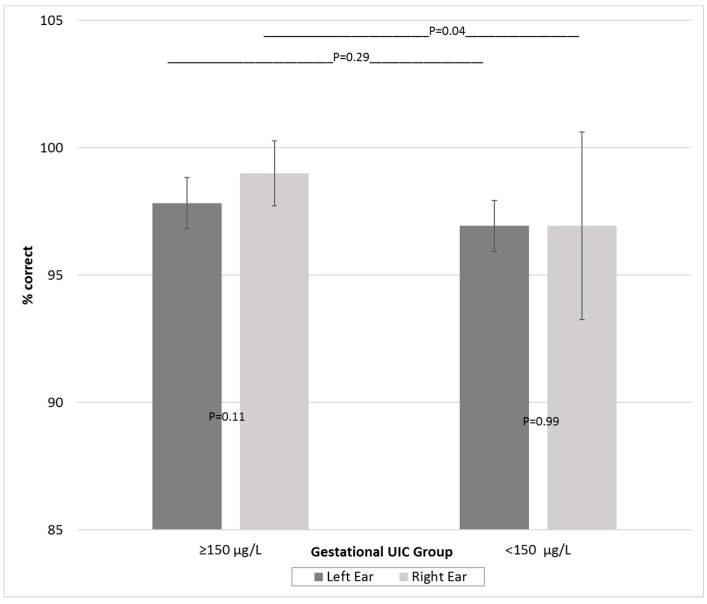
Dichotic digits test (mean and standard deviation): differences within and between UIC groups.

No participants exhibited hearing impairment, with all audiograms classified within normal hearing thresholds. CAPD outcomes are shown in Table 5. The LiSN results indicate that neither group reached Speech Reception Thresholds for clinical indication of a CAPD. Assessment of auditory memory (TAPS-3) showed no difference in the NMF test but poorer performance in the deficient group in the NMR test. The DDT revealed a lower score for the deficient group for the right ear but not the left, compared to the sufficient group, this persisted upon modelling adjustment. A REA was only observed in the sufficient group (Table 5 and Figure 2). The deficient group showed similar scores for both ears, although there was much greater individual variation in right ear performance and poorer performance in both ears compared to the sufficient group.

## 4. Discussion

Our results support the hypothesis that mild GID can lead to long-term adverse consequences for the offspring that are not always ameliorated by adequate iodine nutrition during childhood. The findings build on previous studies (included in reviews by Morreale de Escobar [19], Henrichs [20] and Bath [21] and others) reporting suboptimal neurocognitive outcomes in offspring exposed to mild GID. We demonstrate that some associations between mild GID and literacy outcomes observed at age 9-years [4] persist into adolescence, despite the children having completed more than ten years of formal education and having grown up in an iodine replete environment. Offspring of mothers classified as having deficient iodine nutrition while pregnant had reduced spelling, grammar and reading outcomes that were independent of biological and SES factors known to impact learning. Associations with numeracy and writing were negligible, apart from writing in Year 5 when the task switched from narrative to persuasive writing.

We acknowledge that use of an individual’s UIC to determine their iodine nutrition during pregnancy is problematic, as cut-points for pregnancy (150 μg/L), as for school children (100 μg/L), are validated for population medians, not individuals. Given that UIC is indicative of iodine intake over the past 24 h and may not represent usual levels during pregnancy, misclassification of the severity of ID may occur at the individual level. König et al. [22] suggest that a minimum of ten spot samples are required for UIC to be used to determine an individual’s iodine status; this method, however, is not feasible in most study settings. The UICs in this study are, however, indicative of the iodine status of the individual at the time of measurement. Therefore, a “deficient” (i.e., <150 μg/L) maternal classification indicates that there was at least one period of time during gestation that the fetus was not receiving sufficient iodine. Given the rapidity of neurodevelopmental change during fetal growth, it is feasible that even short periods without sufficient iodine may have adverse consequences. Use of the WHO population-based cut-point of 150 μg/L for pregnant women tends to bias the differences between sufficient and deficient groups towards the null; since those mothers whose true iodine status is close to the cut-point are more likely to be misclassified than those at the extremes. This means that the differences between the groups on NAPLAN outcomes reported here are likely to be underestimated. Additionally, for approximately half of the women iodine status was based on the average of two or three urine samples. Averaging multiple UICs decreases the potential for misclassification. In the absence of a more appropriate individual biomarker, UIC from spot samples has been widely used in studies of maternal iodine nutrition and its impacts on offspring; as such, its use in this study facilitates comparison with other published work.

In addition to the inadequacy of using UIC as a biomarker for iodine nutrition status, our study design has other limitations which may potentially bias the generalizability of the findings. The pregnant women in our study were a volunteer sample from the only public hospital in the southern part of Tasmania and did not include women attending private hospitals. As such, the SES status of our cohort may not be representative of all Tasmanians, with selection bias towards lower SES a possibility. Given, however, that we have previously shown no association between UIC and a range of SES measures in a representative cohort of Tasmanian school children [8,23], we do not believe that any selection bias with respect to SES is influencing our results. Furthermore, even though SES is not associated with the exposure (i.e., maternal iodine status), we have included measures of SES in our models to address the potential for residual confounding given that SES is known to be highly correlated with the outcome measures (i.e., NAPLAN scores). Loss of participants in longitudinal studies also has the potential to introduce bias. However, Table 1 indicates that there are no material differences in the characteristics of those from the original birth cohort who participated in the NAPLAN Study and those who were lost to follow-up.

The NAPLAN results suggest that working memory and auditory processing speed have been impacted by inadequate iodine nutrition in utero. NAPLAN spelling tests both phonographical (auditory pathways) and orthographical (visual pathways) capacity, requiring use of working memory (to hold multiple ideas), combined with fast processing speed (to complete tasks efficiently). Homophone words (e.g. “flower” and “flour”) are used to assess spelling in a task that increases the cognitive load on working memory and processing speed, whereby the first word acts as interference in retrieval of the second word from long-term memory. Other NAPLAN literacy assessments (excluding narrative writing) are similar, requiring students to read and interpret each question, select an appropriate strategy and evaluate their answer before moving onto the next question. While not as cognitively demanding as homophone spelling tasks, these activities place demands on working memory and require an ability to executively process information effectively [24]. In these assessments, information from a previous test item can act as cognitive interference for the current item, adding to the load on working memory.

NAPLAN numeracy, however, requires use of visual processing skills to identify patterns and operational procedural knowledge to complete the task [25]; it does not engage high-level working memory skills or use of auditory pathways. There is also less demand on working memory and processing speed in NAPLAN writing. Unlike the other NAPLAN literacy tests, which consist of a series of unrelated, individual test items, NAPLAN writing is a single item of extended narrative or persuasive writing. As such, students are able to form a schema, which reduces demands on working memory and processing speed. Further support for the role of working memory, as an explanation for the difference in outcomes for the iodine groups, is provided by the large difference for writing in Year 5, when the assessment switched from a narrative to a persuasive task. It is well established that persuasive writing requires greater cognitive effort with respect to working memory, compared to a narrative writing task [26].

While differences between the two iodine groups in spelling persist throughout schooling, the differential in other literacy measures decrease over time resulting in a closing of the gap between the deficient and sufficient groups for grammar and reading. As schooling progresses these NAPLAN literacy tasks become increasingly complex, requiring (in addition to working memory and processing speed) other cognitive processes to successfully complete the tests. The requirement to use additional cognitive processes, which may not have been impacted adversely by GID, could enable the deficient group to “catch up” to the sufficient group over time.

Another consideration is the impact of iodine nutrition during childhood. Brain development does not cease in utero and adequate iodine nutrition during childhood is required for ongoing, optimal neurodevelopment. While gestation of the cohort occurred during a period of mild population ID [8], the cohort have grown up in an iodine replete environment [9,10]. Measurement of UIC in adolescence indicates sufficiency in both groups and its inclusion in NAPLAN models did not alter outcomes. We acknowledge that UIC is not an ideal indicator of an individual’s current or long-term iodine status, nor is it necessarily a reflection of earlier childhood status. However, given adequate adolescence UIC in both groups and stable, adequate population levels throughout childhood in this cohort [9,10], evidence from supplementation studies supports the notion that childhood iodine sufficiency is likely to have had a positive impact of on the cognitive development of both groups. Improvements in hearing [27] and in cognitive tasks requiring perceptual reasoning [28,29] have been reported in children supplemented with iodine. In our cohort, it is probable that sufficient iodine during childhood has resulted in improvements in some of the cognitive processes required to complete the NAPLAN grammar and writing tasks and that this has contributed to closing the gap between the groups.

Given the persisting poorer performance in spelling, however, sufficient iodine in childhood may not correct all deficits resulting from GID. The same supplementation studies did not find improvements in tasks employing working memory and processing speed. Supplementation of mildly-deficient 10–13 year-old New Zealanders, resulted in no improvements in Wechsler Intelligence Scale for Children (WISC) subsets (Letter-Number Sequencing and Symbol Search) assessing working memory and processing speed [28] and, an Albanian study [29] of moderate to severely iodine deficient 10–12 year-olds reported no improvements for the WISC Digit Span test assessing working memory and mixed outcomes for tests of processing speed.

Studies of iodine supplementation during pregnancy also provide evidence that mild ID can have adverse neurodevelopmental consequences for the offspring. Velasco [30] reported infants, whose mothers did not receive first trimester iodine supplementation, had lower Psychomotor Development Index and Behavior Rating Scale scores (Bayley Scales of Infant Development). Berbel [31] reported significantly delayed neurobehavioral performance (Brunet-Lézine Scale) in children of mothers without first trimester supplementation. In contrast to these two unrandomized studies, a recent randomized controlled trial of iodine supplementation of pregnant women (200 μg of iodine daily versus placebo) in India and Thailand failed to find differences in verbal IQ (Wechsler Preschool and Primary Scale of Intelligence Third Edition), or in other measures of IQ and executive function in the children at age 5–6 years [32]. We concur with Bath’s commentary [33] that there are a number of factors that may have contributed to the lack of effect observed in this study. First, although supplementation began at or before 14 weeks gestation, this may have been too late to correct for any neurological damage from ID that may have occurred in both groups earlier in the first trimester. Adequate maternal iodine in early pregnancy is crucial for fetal neurodevelopment [1] and there is increasing evidence that adequate maternal thyroid stores prior to pregnancy are also important [6]. Second, although the randomized groups were classified at baseline as mildly iodine deficient (median UIC: iodine supplemented group 134 μg/L; placebo group 125 μg/L) the levels are much higher than the median value of our maternal cohort (UIC 83.2 μg/L) and those of the UK study (91 μg/L) [3]. Given that UIC is measured along a continuum and baseline levels in the supplemented and placebo groups are closer to the 150 μg/L cut-point for sufficiency, the results of the neurodevelopmental testing will tend towards a null finding. This movement towards the null is further exacerbated by a return to iodine sufficiency in the second and third trimesters of not only the supplemented group but also the placebo group. Although the supplemented group has statistically significantly higher UICs than the placebo group, both are greater than 150 μg/L cut-point and therefore classified as iodine sufficient.

In our study, the persistent differences in spelling, coupled with lack of improvement in supplementation studies of tasks requiring working memory and processing speed, indicate that mild GID is impacting at a stage and/or on a specific element of neurodevelopment that is very resistant to later change. GID can cause irreversible abnormalities to fetal neuronal cytoarchitecture and morphology; alter normal neuronal proliferation and migration; affect usual densities of dendrites and; reduce myelination of axons [34]. Rodent studies highlight the important role of thyroid hormones in laying down neurofilaments upon which myelination occurs [34,35]. If insufficient maternal iodine compromises neurofilament and other cytostructural development, even ongoing myelination during later development may not be able to fully compensate for earlier structural impairment and resultant slower processing speeds. These notions are supported by evidence that myelination, particularly of the corpus callosum (CC), is incomplete at birth and continues through childhood and into early adolescence and that some children with learning difficulties may have incomplete CC myelination or take longer for myelination to be complete [36]. Adequate childhood iodine may act to reduce, but not eliminate, the differences resulting from GID. We suggest that despite ongoing myelination in both groups (facilitated via adequate childhood iodine), structural deficits in the deficient iodine group (as a consequence of GID) prevent optimal myelination, which in turn results in deficits in processing speed that manifest as persisting reductions in spelling outcomes.

The CELF-4 and CAPD study was designed to explore possible mechanisms for the observed association between mild GID and reduced literacy outcomes. We acknowledge that interpretation of this sub-study requires caution, given the potential bias due to the higher SES of the participants and the earlier gestational age of UIC measurement. Nevertheless, the results support the notion that deficits in working memory and processing speed are potential drivers of reduced literacy for those impacted by mild GID. Further studies will be required to confirm these preliminary observations.

Reduced outcomes in all CELF-4 subtests and indexes in the iodine deficient group is indicative of a processing delay and likely reflects detection of memory difficulties resulting from the demands of some subtest tasks. The CELF-4 authors state that “many of the test items require the child to hold several items in short-term memory at once, then compare/analyze them and come up with a right answer” [15]. The FS subtest, for example, requires self-generation of complete, grammatically correct and meaningful spoken sentences of increasing length and complexity about a visual stimuli using a targeted word or phrase—a task requiring high level working memory skills (in addition to auditory and visual processing).

The TAPS-3 NMF subtest measures short-term memory capacity and memory span (i.e., storing but not manipulating information), whereas NMR uses working memory to simultaneously store information and perform cognitive tasks where attention is divided and short-term memory capacity is limited (i.e., storing and manipulating information) [37]. Poorer NMR, but not NMF, performance in the deficient group is evidence of reduced working memory capacity. Difficulties with digital span memory and saying numbers backwards have been identified in children with reading problems [38]. Similarly, a study of adolescents found no association between maternal thyroxine (T_4_) or triiodothyronine (T_3_) levels and a forward memory span test employing short-term memory but positive associations between T_4_ and backward memory and serial position tasks requiring an ability to store and manipulate information [39].

The lack of a REA and poorer performance in both ears in the deficient group, in the DTT, adds support to the concept that mild GID is impacting fetal neuronal cytoarchitecture and morphology, particularly in the CC. This is contrary to Kimura’s model [40] which suggests that any structural deficits in neuronal cytoarchitecture, particularly the CC, in the deficient group would lead to increased REA, as scores for the left ear would decrease. Indeed, there is an abundance of literature reporting increased REA in children with a range of language and learning difficulties. We suggest, however, that the results for the deficient group are more akin to those observed in individuals with congenital callosal agenesis who are unable to use contralateral paths from the left ear via the CC to the left temporal language lobe in DTTs [41]. Westerhausen [41] states, “a developing brain possesses sufficient structural plasticity to compensate, at least to some degree, the congenital lack of callosal connections” and suggests that lack of REA may result from increased use of the usually weaker ipsilateral pathways. It is plausible that, in our deficient group, the more direct ipsilateral pathways from left ear to left temporal lobe are just as effective as the contralateral pathways via the structurally impaired CC; alternatively, the left ear ipsilateral pathways may compensate for deficits in CC integrity and these are used in preference to usual left ear contralateral paths. Both may explain the lack of REA and suggest there is no difference in the processing speed between the left and right ears of the deficient group. The existence of possible structural deficits in the CC of the deficient group may explain the reduced DDT outcomes.

## 5. Conclusions

Our findings support previous research indicating that even mild GID can negatively impact fetal neurodevelopment. We have shown that reductions in educational outcomes associated with mild GID endure and are not fully ameliorated by iodine sufficiency during childhood. That a group of adolescents should have persisting poorer performance and continue to lag behind their peers with respect to language and literacy development, 15 years after experiencing mild GID and despite completing ten years of schooling in an iodine replete environment, points to neurological damage occurring in utero that is resistant to change. The findings have important public health implications for pregnant women. Despite mandatory fortification and a recommendation for daily iodine supplementation during pregnancy [42], many Australian women remain mildly iodine deficient during pregnancy [43,44]. Action is required to eliminate this preventable condition and ensure that no more children are prevented from reaching their full cognitive potential.

## Figures and Tables

**Table 1:** Characteristic comparisons of participants and non-participants: Original birth cohort (*n* = 421), National Assessment Program–Literacy and Numeracy (NAPLAN) Study (*n* = 266) and Comprehensive Evaluation of Language Fundamentals (CELF-4) and Central Auditory Processing Disorder (CAPD) Study (*n* = 45).

	1. Original Cohort (*n* = 421)	*n*	2a. NAPLAN Study NOT Followed-Up (*n* = 155)	*n*	2b. NAPLAN Participants (*n* = 266)	*n*	*p* Value ^5^ 2a vs. 2b	3a. CELF-4/CAPD Study NOT Followed-Up (*n* = 221)	*n*	3b. CELF-4/CAPD Study Participants (*n* = 45)	*n*	*p* Value ^5^ 3a vs. 3b
**Gestational Measures**												
Maternal age at birth of child (years)	28.3 (5.6)	419	28.6 (5.2)	153	28.1 (5.9)	266	0.374	27.7 (5.8)	221	30.0 (5.9)	45	0.021
Gestational Length (weeks)	39.2 (2.1)	412	39.1 (2.4)	148	39.2 (1.9)	264	0.744	39.1 (1.9)	219	39.7 (1.0)	45	0.061
Preterm birth (<37 weeks, %)	8.0%	33/412	8.1%	12/148	8.0%	21/264	0.956	9.1%	20/219	0.0%	0/45	0.035
Birth Weight (g)	3440 (539)	411	3469 (462)	148	3423 (578)	263	0.404	3383 (584)	219	3623 (509)	44	0.012
Low birth weight (<2500 g, %)	5.1%	21/411	2.7%	4/148	6.5%	17/263	0.097	7.8%	17/219	0.0%	0/44	0.056
Male sex (%)	47.6%	199/418	48.0%	73/153	47.4%	126/266	0.897	48.0%	106/221	44.4%	20/45	0.668
Gestational age at time of UI ^1^ collection (weeks)	23.6 (9.7)	421	23.5 (9.7)	155	23.7 (9.7)	266	0.848	25.2 (9.7)	221	16.0 (4.6)	45	<0.0001
Maternal UIC (μg/L)	86.0 (50.0–192.3)	421	94.0 (53.0–228.3)	155	83.2 (46.0–180.0)	266	0.077	81 (46.0–168.0)	221	99 (52.5–210.0)	45	0.391
Maternal UIC ≥ 150 μg/L (%)	33.0%	139/421	36.8%	57/155	30.8%	82/266	0.211	30.3%	67/221	33.3%	15/45	0.691
**Socio-Economic Measures**												
Maternal education (>year 10, %) ^2^					69.9%	181/259		67.9%	146/215	79.6%	35/44	0.125
Maternal occupation (%) ^3^					21.7%	53/244		19.6%	40/204	32.5%	13/40	0.071
Paternal education (>year 10, %) ^2^					70.1%	157/224		68.1%	126/185	79.5%	31/39	0.158
Paternal occupation (%) ^3^					27.4%	57/208		24.4%	42/172	41.7%	15/36	0.035
Indigenous status (%) ^4^					12.3%	32/260		12.5%	27/216	11.4%	5/44	0.843
**Year 3 NAPLAN**												
Spelling Score					386.7 (93.4)	248		381.6 (94.8)	203	409.5 (83.8)	45	0.070
Grammar Score					390.3 (102.1)	248		384.0 (102.7)	203	418.3 (95.5)	45	0.041
Reading Score					397.3 (91.4)	247		395.0 (90.9)	202	407.7 (93.8)	45	0.399
Writing Score					400.4 (72.2)	245		398.1 (73.3)	200	410.6 (67.2)	45	0.295
Numeracy Score					380.4 (81.1)	241		373.6 (80.2)	197	410.8 (79.1)	44	0.006

Data are means (standard deviations (SD)) for continuous variables and percentages (*n*/*N* %) for categorical variables. Median and interquartile range (IQR) shown for maternal urinary iodine concentration (UIC). ^1^ UI—Urinary Iodine. ^2^ Level of education divided into two categories: Completed Year 10 or below vs. Completed higher than Year 10. The percentage in the latter group is given. ^3^ Occupation was divided into two categories: Unemployed/manual vs. Professional/paraprofessional/managers. The percentage in the latter group is given. ^4^ Indigenous status classified into two categories: Yes, identifies as Aboriginal or Torres Strait Islander or both, vs. No, does not identify as Aboriginal or Torres Strait Islander or both. The percentage of the former group is given. ^5^ *p* values were calculated using Student’s *t*-test or Mann-Whitney *U*-test for continuous variables and *Χ*^2^ tests for categorical variables.

**Table 2:** Characteristics of participants by maternal UIC during pregnancy (≥150 vs. <150 μg/L): data for children in NAPLAN study (*n* = 266) and CELF-4/CAPD study (*n* = 45).

	NAPLAN Study					CELF-4/CAPD Study				
	UIC ≥ 150 μg/L “Sufficient”	*n*	UIC < 150 μg/L “Deficient”	*n*	*p* Value ^4^	UIC ≥ 150 μg/L “Sufficient”	*n*	UIC < 150 μg/L “Deficient”	*n*	*p* Value ^4^
**Gestational & Birth Measures**										
Maternal age at birth of child (years)	28.1 (5.5)	82	28.1 (6.1)	184	0.981	29.3 (6.3)	15	30.3 (5.8)	30	0.584
Gestational Length (weeks)	39.3 (1.8)	81	39.2 (1.8)	183	0.747	39.7 (1.0)	15	39.7 (1.1)	30	0.947
Preterm birth (<37 weeks, %)	9.9%	8/81	6.6%	12/183	0.347	0.0%	0/15	0.0%	0/30	-
Birth Weight (g)	3399 (550)	82	3434 (592)	181	0.656	3644 (426)	15	3612 (554)	29	0.847
Low birth weight (<2500 g, %)	7.3%	6/82	6.1%	11/181	0.705	0.0%	0/15	0.0%	0/29	-
Male sex (%)	41.5%	34/82	50.0%	92/184	0.198	40.0%	6/15	46.7%	14/30	0.671
Gestational age at time of UI collection (week)	23.3 (9.6)	82	23.8 (9.7)	184	0.649	15.7 (3.8)	15	16.2 (5.0)	30	0.720
**Socio-Economic Status Measures** (when starting school)										
Maternal education (>year 10, %) ^1^	66.3%	53/80	71.2%	128/179	0.394	66.7%	10/15	86.2%	25/29	0.128
Maternal occupation (%) ^2^	20.0%	15/75	22.5%	38/169	0.664	30.8%	4/13	33.3%	9/27	0.871
Paternal education (>year 10, %) ^1^	73.5%	50/68	68.6%	107/156	0.458	92.3%	12/13	73.1%	19/26	0.161
Paternal occupation (%) ^2^	31.8%	20/63	25.5%	37/145	0.355	33.3%	4/12	45.8%	11/24	0.473
Indigenous status (%) ^3^	11.4%	9/79	12.7%	23/181	0.767	14.3%	2/14	10.0%	3/30	0.677
**Adolescent UIC** (aged 13–14 years)						159.5 (103.0–231.0)	14	150 (109.0–179.0)	30	0.597
**NAPLAN**										
Spelling Score										
Year 3	414.9 (109.2)	72	375.2 (83.7)	176	0.002	462.0 (71.8)	15	383.2 (77.6)	30	0.002
Year 5	483.4 (78.7)	79	452.6 (78.4)	173	0.004	512.2 (56.8)	15	447.7 (75.9)	29	0.006
Year 7	534.6 (86.9)	65	504.9 (74.5)	146	0.012	574.4 (56.1)	14	498.1 (74.9)	24	0.002
Year 9	575.3 (91.0)	61	545.4 (70.9)	133	0.014	601.2 (89.7)	14	533.0 (82.1)	21	0.027
Grammar Score										
Year 3	408.7 (100.2)	72	382.7 (102.2)	176	0.068	464.8 (56.6)	15	395.1 (103.0)	30	0.019
Year 5	476.0 (93.3)	79	454.3 (83.1)	173	0.066	540.8 (81.3)	15	461.1 (92.2)	29	0.007
Year 7	522.4 (89.2)	65	498.0 (84.5)	146	0.070	565.7 (52.7)	14	484.7 (93.1)	24	0.005
Year 9	559.2 (72.1)	61	543.8 (60.8)	133	0.124	561.4 (53.2)	14	531.3 (61.9)	21	0.145
Reading Score										
Year 3	417.8 (96.1)	71	389.0 (88.3)	176	0.025	462.1 (86.8)	15	380.5 (86.1)	30	0.005
Year 5	479.4 (88.3)	79	459.3 (83.7)	174	0.083	540.6 (77.5)	15	455.7 (88.6)	29	0.003
Year 7	528.4 (80.2)	64	515.3 (72.8)	143	0.248	558.3 (60.7)	13	510.2 (73.0)	24	0.051
Year 9	575.8 (80.2)	60	558.9 (63.2)	126	0.120	596.3 (63.4)	14	557.6 (71.7)	20	0.115
Writing Score										
Year 3	407.6 (70.8)	72	397.4 (72.8)	173	0.314	445.6 (54.6)	15	393.1 (66.9)	30	0.012
Year 5	470.4 (68.9)	79	448.1 (72.6)	172	0.022	522.6 (70.6)	15	476.5 (69.1)	29	0.043
Year 7	484.6 (102.6)	65	477.9 (67.3)	144	0.574	538.0 (63.6)	14	482.7 (59.5)	24	0.011
Year 9	530.0 (105.7)	61	529.5 (69.9)	132	0.969	540.9 (48.4)	14	541.4 (90.2)	21	0.986
Numeracy Score										
Year 3	384.9 (84.6)	69	378.6 (79.9)	172	0.583	432.1 (72.8)	14	400.9 (81.1)	30	0.228
Year 5	465.2 (69.1)	78	457.7 (65.4)	175	0.412	490.5 (41.4)	15	469.0 (57.0)	29	0.203
Year 7	520.4 (66.6)	61	508.9 (59.0)	146	0.219	545.1 (56.8)	12	511.7 (61.3)	23	0.127
Year 9	562.5 (63.8)	63	558.6 (53.1)	130	0.654	579.7 (39.3)	14	564.3 (57.1)	20	0.390

Data are means (SD) for continuous variables and percentages (*n*/*N* %) for categorical variables. Median and interquartile range (IQR) shown for maternal and adolescent UIC. ^1^ Level of education divided into two categories: Completed Year 10 or below vs. Completed higher than Year 10. The percentage in the latter group is given. ^2^ Occupation was divided into two categories: Unemployed/manual vs. Professional/paraprofessional/managers. The percentage in the latter group is given. ^3^ Indigenous status classified into two categories: Yes, identifies as Aboriginal or Torres Strait Islander or both, vs. No, does not identify as Aboriginal or Torres Strait Islander or both. The percentage of the former group is given. ^4^ *p* values were calculated using Student’s *t*-test or Mann-Whitney *U*-test for continuous variables and *Χ*^2^ tests for categorical variables. Please note: Total numbers of children with specific NAPLAN outcomes always less than the *n* = 266 cohort total; n varies depending upon the number of children absent on each testing day.

**Table 3:** (**a**) Examination of NAPLAN associations with maternal urinary iodine concentration (UIC) as a continuous variable, *n* = 266 ^1^. (**b**) NAPLAN mixed-effects multiple imputation using chained equations (MICE) ^2^ models: Differences in NAPLAN outcomes for offspring of mothers with deficient UIC (<150 μg/L) during pregnancy compared to mothers with sufficient UIC (≥150 μg/L), *n* = 266.

NAPLAN	School Year of Test	(a) UIC Continuous			(b) UIC Categorical (<150 μg/L vs. ≥150 μg/L)							
		β Coefficient	*p* Value	*n*	Unadjusted		Adjusted for Biological Factors ^3^		Adjusted for Biological & Socioeconomic Factors ^4^		Adjusted for Biological & Socioeconomic Factors & Adolescent UIC ^5^	
					β (95% CI) ^6^	*p* Value	β (95% CI)	*p* Value	β (95% CI)	*p* Value	β (95% CI)	*p* Value
Spelling	Year 3	0.043	0.011	248	−43.6 (−65.9 to −21.4)	<0.0001	−43.4 (−65.3 to −21.5)	<0.0001	−44.4 (−66.2 to −22.5)	<0.001	−41.4 (−65.1 to −17.6)	0.001
	Year 5	0.040	0.002	252	−34.1 (−56.2 to −11.9)	0.003	−34.1 (−55.8 to −12.4)	0.002	−35.0 (−56.6 to −13.4)	0.002	−31.9 (−55.4 to −8.4)	0.008
	Year 7	0.042	0.002	211	−35.1 (−57.4 to −12.7)	0.002	−35.0 (−56.9 to −13.1)	0.002	−35.9 (−57.7 to −14.2)	0.001	−32.6 (−56.5 to −8.8)	0.008
	Year 9	0.037	0.005	194	−33.5 (−56.8 to −10.1)	0.005	−33.6 (−56.5 to −10.7)	0.004	−34.8 (−57.6 to −12.0)	0.003	−31.6 (−57.0 to −6.2)	0.015
Grammar	Year 3	0.027	0.150	248	−24.2 (−47.7 to −0.7)	0.043	−23.3 (−46.4 to −0.3)	0.047	−24.2 (−47.2 to −1.3)	0.039	−26.2 (−50.0 to −2.3)	0.031
	Year 5	0.035	0.015	252	−28.3 (−51.6 to −5.0)	0.017	−26.9 (−49.8 to −4.0)	0.021	−28.0 (−50.8 to −5.2)	0.016	−29.6 (−53.5 to −5.7)	0.015
	Year 7	0.036	0.016	211	−24.8 (−48.9 to −0.8)	0.043	−23.8 (−47.3 to −0.3)	0.047	−24.9 (−48.3 to −1.5)	0.037	−26.3 (−50.3 to −2.3)	0.057
	Year 9	0.032	0.003	194	−13.8 (−37.7 to +10.1)	0.256	−13.1 (−36.8 to +10.6)	0.278	−13.9 (−37.3 to +9.6)	0.246	−15.7 (−40.2 to +8.9)	0.211
Reading	Year 3	0.042	0.013	247	−30.2 (−52.1 to −8.3)	0.007	−29.4 (−51.0 to −7.8)	0.008	−30.5 (−51.9 to −9.1)	0.005	−29.4 (−51.6 to −7.2)	0.010
	Year 5	0.022	0.115	253	−23.8 (−45.4 to −2.1)	0.031	−22.7 (−43.9 to −1.5)	0.036	−23.9 (−44.8 to −2.9)	0.026	−22.8 (−44.4 to −1.2)	0.039
	Year 7	0.031	0.018	207	−14.0 (−36.8 to +8.7)	0.226	−13.2 (−35.5 to +9.1)	0.246	−14.2 (−36.3 to +7.8)	0.205	−13.2 (−35.6 to +9.2)	0.249
	Year 9	0.028	0.017	186	−15.4 (−38.0 to +7.2)	0.181	−14.5 (−36.7 to +7.6)	0.198	−15.6 (−37.4 to +6.3)	0.163	−14.5 (−36.8 to +7.8)	0.202
Writing	Year 3	0.017	0.216	245	−11.2 (−32.2 to +9.9)	0.297	−7.6 (−27.7 to +12.5)	0.459	−8.3 (−28.4 to +11.7)	0.415	−9.5 (−30.6 to +11.6)	0.377
	Year 5	0.029	0.016	251	−25.5 (−46.1 to −4.9)	0.015	−22.1 (−41.9 to −2.3)	0.029	−22.9 (−42.6 to −3.1)	0.023	−24.0 (−44.9 to −3.2)	0.024
	Year 7	0.025	0.070	209	−7.9 (−30.9 to +15.1)	0.498	−3.7 (−26.0 to +18.6)	0.746	−4.1 (−26.3 to +18.1)	0.717	−5.6 (−29.7 to +18.5)	0.647
	Year 9	0.030	0.036	193	+1.2 (−23.4 to +25.7)	0.927	+4.0 (−19.7 to +27.6)	0.741	+3.5 (−20.2 to +27.2)	0.772	+2.1 (−22.4 to +26.5)	0.869
Numeracy	Year 3	0.017	0.269	241	−9.6 (−28.1 to +9.0)	0.314	−9.8 (−28.2 to +8.5)	0.292	−10.7 (−28.9 to +7.5)	0.248	−11.7 (−32.2 to +14.8)	0.263
	Year 5	0.028	0.011	253	−9.7 (−27.6 to +8.3)	0.291	−10.2 (−28.0 to +7.6)	0.262	−11.1 (−28.7 to +6.6)	0.221	−12.0 (−31.8 to +7.8)	0.235
	Year 7	0.022	0.041	207	−12.3 (−30.4 to +5.8)	0.183	−12.7 (−30.6 to +5.1)	0.163	−13.6 (−31.4 to +4.2)	0.133	−14.6 (−34.3 to +5.2)	0.149
	Year 9	0.022	0.025	193	−9.0 (−27.6 to +9.6)	0.343	−9.6 (−28.0 to +8.9)	0.308	−10.4 (−28.7 to +7.8)	0.263	−11.4 (−31.0 to +8.3)	0.255

^1^ Please note: Total numbers of children with specific NAPLAN outcomes always less than the *n* = 266 cohort total; n varies depending upon the number of children absent on each testing day. ^2^ Variables included in imputation model: regress (NAPLAN outcomes at each time point, birth weight, gestational length, adolescent UIC, Tasmanian State Government Student Assessment and Reporting Information scores for English and Maths in Years 2, 3 and 4, Australian Bureau of Statistics Socio-economic Indexes for Areas (scores): Index of Relative Socio-economic Advantage and Disadvantage, Index of Relative Socio-economic Disadvantage, Index of Economic Resources and, Index of Education and Occupation); ologit (paternal education); pmm (maternal education, low birth weight, preterm birth) and ; complete (maternal age at birth of child, sex of child, gestational age at time of maternal UI collection). ^3^ Adjusted for gestational age at the time of maternal UI collection, maternal age at birth of child, gestational length at time of birth, birth weight and sex. ^4^ Adjusted for all of the above and for maternal education. ^5^ Adjusted for all of the above and for adolescent UIC. ^6^ CI—Confidence Interval.

**Table 4:** CELF-4: Differences in outcomes ^1^ and regression models for offspring of mothers with deficient UIC (<150 μg/L) (*n* = 15) during pregnancy compared to mothers with sufficient UIC (≥150 μg/L) (*n* = 30).

	UIC ≥ 150 μg/L	UIC < 150 μg/L	*p* Value ^5^	Unadjusted		Adjusted for Biological Factors ^2^		Adjusted for Biological & Socioeconomic Factors ^3^		Adjusted for Biological & Socioeconomic Factors & Adolescent UIC ^4^	
				β (95% CI)	*p* Value	β (95% CI)	*p* Value	β (95% CI)	*p* Value	β (95% CI)	*p* Value
**Indexes (Standard Scores)**											
Core Language Index (RS, FS, WC-T & WD) ^6^	101.5 (12.0)	94.3 (14.5)	0.106	−7.2 (−16.0 to 1.6)	0.106	−7.1 (−15.6 to 1.5)	0.101	−7.9 (−16.9 to 1.1)	0.085	−8.6 (−18.2 to 1.0)	0.076
Receptive Language Index (WC-R, USP & SR) ^6^	101.1 (9.5)	96.9 (15.5)	0.352	−4.1 (−13.0 to 4.7)	0.352	−4.4 (−12.9 to 4.2)	0.307	−4.9 (−13.9 to 4.1)	0.279	−5.8 (−15.5 to 4.0)	0.236
Expressive Language Index (RS, FS & WC-E) ^6^	103.3 (11.5)	95.2 (14.6)	0.069	−8.1 (−16.9 to 0.7)	0.069	−7.6 (−16.4 to 1.1)	0.085	−8.5 (−17.6 to 0.7)	0.069	−8.9 (−18.7 to 1.0)	0.077
Language Content Index (WD, USP & SA) ^6^	102.5 (11.0)	96.9 (15.9)	0.231	−5.6 (−14.8 to 3.7)	0.231	−5.7 (−14.4 to 2.9)	0.186	−7.0 (−15.9 to 1.9)	0.121	−7.8 (−17.2 to 1.6)	0.103
Language Memory Index (RS, FS & SR) ^6^	100.6 (11.0)	94.7 (15.4)	0.193	−5.9 (−14.9 to 3.1)	0.193	−4.9 (−13.6 to 3.9)	0.271	−5.3 (−14.6 to 4.0)	0.254	−5.8 (−15.9 to 4.3)	0.249
Working Memory Index (NR-T & FSq1) ^6^	96.8 (10.9)	90.7 (15.1)	0.169	−6.1 (−15.0 to 2.7)	0.169	−6.4 (−15.3 to 2.5)	0.154	−4.8 (−13.8 to 4.3)	0.292	−5.5 (−15.3 to 4.2)	0.258
**Sub-tests (Scaled Scores)**											
Recalling Sentences (RS)	10.07 (2.15)	9.27 (2.79)	0.336	−0.8 (−2.5 to 0.9)	0.336	−0.5 (−2.1 to 1.1)	0.532	−0.7 (−2.4 to 0.9)	0.374	−0.6 (−2.4 to 1.2)	0.477
Formulated Sentences (FS)	10.73 (2.43)	8.93 (3.11)	0.057	−1.8 (−3.7 to 0.1)	0.057	−1.7 (−3.5 to 0.2)	0.075	−1.7 (−3.6 to 0.3)	0.091	−1.8 (−3.9 to 0.3)	0.087
Word Classes—Receptive (WC-R)	9.40 (2.26)	8.86 (2.77)	0.521	−0.5 (−2.2 to 1.1)	0.521	−0.7 (−2.3 to 0.9)	0.396	−0.8 (−2.5 to 0.9)	0.366	−0.8 (−2.6 to 1.1)	0.392
Word Classes—Expressive (WC-E)	10.73 (2.76)	9.69 (3.64)	0.336	−1.0 (−3.2 to 1.1)	0.336	−1.2 (−3.4 to 1.0)	0.268	−1.4 (−3.7 to 0.9)	0.224	−1.6 (−4.1 to 0.8)	0.190
Word Classes—Total (WC-T)	9.93 (2.74)	9.24 (3.28)	0.488	−0.7 (−2.7 to 1.3)	0.488	−0.9 (−2.8 to 1.1)	0.389	−1.0 (−3.1 to 1.1)	0.358	−1.1 (−3.4 to 1.1)	0.314
Word Definitions (WD)	10.13 (3.20)	9.40 (3.46)	0.496	−0.7 (−2.9 to 1.4)	0.496	−0.8 (−2.9 to 1.3)	0.430	−1.1 (−3.3 to 1.1)	0.330	−1.4 (−3.7 to 0.9)	0.221
Understanding Spoken Paragraphs (USP)	11.40 (2.47)	10.67 (3.29)	0.451	−0.7 (−2.7 to 1.2)	0.451	−0.8 (−2.6 to 1.1)	0.400	−1.0 (−2.8 to 0.8)	0.279	−1.2 (−3.1 to 0.8)	0.247
Sentence Assembly (SA)	9.33 (2.61)	8.07 (2.75)	0.146	−1.3 (−3.0 to 0.5)	0.146	−1.3 (−2.9 to 0.4)	0.131	−1.4 (−3.1 to 0.3)	0.113	−1.3 (−3.1 to 0.6)	0.161
Semantic Relationships (SR)	9.53 (1.77)	8.83 (3.12)	0.426	−0.7 (−2.5 to 1.1)	0.426	−0.7 (−2.4 to 1.1)	0.443	−0.6 (−2.4 to 1.2)	0.513	−0.9 (2.9 to 1.1)	0.360
Number Repetition—Forwards (NR-F)	9.07 (2.38)	8.20 (2.47)	0.267	−0.9 (−2.4 to 0.7)	0.267	−0.9 (−2.5 to 0.6)	0.229	−1.1 (−2.7 to 0.5)	0.187	−1.4 (−3.1 to 0.4)	0.116
Number Repetition—Backwards (NR-B)	9.67 (1.50)	9.60 (3.11)	0.938	−0.1 (−1.8 to 1.7)	0.938	−0.1 (−1.9 to 1.7)	0.926	−0.1 (−1.8 to 1.9)	0.947	+0.1 (−1.9 to 2.1)	0.946
Number Repetition—Total (NR-T)	9.20 (1.78)	8.47 (2.93)	0.380	−0.7 (−2.4 to 0.9)	0.380	−0.8 (−2.5 to 0.9)	0.338	−0.8 (−2.5 to 1.0)	0.363	−1.0 (−2.8 to 0.9)	0.308
Familiar Sequences 1 (FSq1)	9.67 (2.55)	8.53 (2.96)	0.213	−1.3 (−2.9 to 0.7)	0.212	−1.2 (−3.0 to 0.6)	0.188	−0.7 (−2.4 to 1.1)	0.448	−0.7 (−2.6 to 1.2)	0.456

^1^ Data for differences in outcomes are means (SD) for continuous variables and percentages (*n*/*N* %) for categorical variables. ^2^ Model adjusted for gestational age at the time of maternal UI collection, maternal age at birth of child, gestational length at time of birth, birth weight and sex. ^3^ Model adjusted for all of the above and for maternal education. ^4^ Model adjusted for all of the above and for adolescent UIC. ^5^ *p* values for differences in outcomes were calculated using *t* tests for continuous variables and *Χ*^2^ tests for categorical variables. ^6^ RS—Recalling Sentences; FS—Formulated Sentences; WC-T—Word Classes-Total; WC-R—Word Classes-Receptive; USP—Understanding Spoken Paragraphs; SR—Semantic Relationships; WC-E—Word Classes-Expressive; SA—Sentence Assembly; NR-T—Number Repetition-Total; FSq1—Familiar Sequences 1.

**Table 5:** CAPD: Differences in outcomes ^1^ and regression models for offspring of mothers with deficient UIC (<150 μg/L) (*n* = 15) during pregnancy compared to mothers with sufficient UIC (≥150 μg/L) (*n* = 30).

	UIC ≥ 150 μg/L	UIC < 150 μg/L		Unadjusted		Adjusted for Biological Factors ^2^		Adjusted for Biological & Socioeconomic Factors ^3^		Adjusted for Biological & Socioeconomic Factors & Adolescent UIC ^4^	
			*p* Value ^5^	β (95% CI)	*p* Value	β (95% CI)	*p* Value	β (95% CI)	*p* Value	β (95% CI)	*p* Value
**LiSN ^6^**											
SRT(dB) High Cue:											
different voices, different direction (dv90)	−15.6 (1.7)	−16.3 (1.8)	0.230	−0.7 (−1.8 to 0.4)	0.229	−0.7 (−1.8 to 0.5)	0.263	−0.5 (−1.7 to 0.7)	0.408	−0.4 (−1.7 to 0.9)	0.517
same voices, diff direction (sv90)	−14.2 (1.8)	−14.7 (1.9)	0.371	−0.5 (−1.7 to 0.6)	0.371	−0.6 (−1.8 to 0.6)	0.310	−0.5 (−1.7 to 0.8)	0.458	−0.2 (−1.5 to 1.1)	0.727
diff voices, same direction (dv0)	−6.3 (2.1)	−7.2 (2.9)	0.298	−0.9 (−2.6 to 0.8)	0.298	−0.8 (−2.5 to 1.0)	0.387	−0.7 (−2.5 to 1.1)	0.462	−0.5 (−2.5 to 1.5)	0.607
SRT(dB) Low Cue:											
same voices, same direction (sv0)	−1.5 (1.0)	−1.3 (1.6)	0.682	0.2 (−0.7 to 1.1)	0.682	0.3 (−0.6 to 1.2)	0.539	0.3 (−0.6 to 1.3)	0.469	0.4 (−0.7 to 1.4)	0.477
Advantage Measures (dB) ^7^:											
Talker Advantage (dv0–sv0)	5.2 (2.3)	5.9 (2.5)	0.393	0.7 (−0.9 to 2.2)	0.393	0.7 (−0.9 to 2.3)	0.375	0.7 (−0.9 to 2.4)	0.377	0.6 (−1.2 to 2.4)	0.528
Spatial Advantage (sv90–sv0)	12.7 (1.5)	13.4 (1.9)	0.208	0.7 (−0.4 to 1.8)	0.208	0.9 (−0.3 to 2.0)	0.128	0.8 (−0.4 to 2.0)	0.186	0.6 (−0.7 to 1.9)	0.353
Total Advantage (dv90–sv0)	14.1 (1.6)	15.0 (1.9)	0.140	0.9 (−0.3 to 2.0)	0.140	0.9 (−0.3 to 2.1)	0.127	0.8 (−0.4 to 2.1)	0.188	0.8 (−0.6 to 2.1)	0.249
**TAPS-3 ^8^**											
Number Memory Forward (NMF)	8.3 (3.3)	8.7 (3.0)	0.708	0.4 (−1.6 to 2.3)	0.708	0.4 (−1.7 to 2.5)	0.678	0.5 (−1.7 to 2.7)	0.658	0.7 (−1.6 to 3.0)	0.556
Number Memory Reversed (NMR)	9.7 (2.3)	8.8 (2.3)	0.188	−1.0 (−2.4 to 0.5)	0.188	−1.0 (−2.5 to 0.4)	0.153	−1.1 (−2.6 to 0.5)	0.162	−1.1 (−2.7 to 0.5)	0.184
**Dichotic Digits**											
Right Ear	99.0 (1.3)	96.9 (3.7)	0.043	−2.1 (−4.1 to −0.1)	0.043	−2.0 (−3.9 to 0.0)	0.048	−2.2 (−4.2 to −0.2)	0.035	−2.2 (−4.4 to 0.0)	0.054
Left Ear	97.8 (2.3)	96.9 (2.8)	0.293	−0.9 (−2.6 to 0.8)	0.293	−0.7 (−2.2 to 0.9)	0.391	−0.7 (−2.3 to 1.0)	0.422	−1.0 (−2.7 to 0.8)	0.271
Right Ear Advantage (REA) (Right-Left Ear)	1.17 (2.65)	0.01 (3.14)	0.227	−1.2(−3.1 to 0.8)	0.227	−1.3 (−3.3 to 0.8)	0.212	−1.5 (−3.7 to 0.6)	0.156	−1.2 (−3.5 to 1.1)	0.295
Right Handed	78.6% (11/14)	82.8% (24/29)	0.741								

^1^ Data for differences in outcomes are means (SD) for continuous variables and percentages (*n*/*N* %) for categorical variables; ^2^ Model adjusted for gestational age at the time of maternal UI collection, maternal age at birth of child, gestational length at time of birth, birth weight and sex; ^3^ Model adjusted for all of the above and for maternal education; ^4^ Model adjusted for all of the above and for adolescent UIC; ^5^ *p* values for differences in outcomes were calculated using *t* tests for continuous variables and *Χ*^2^ tests for categorical variables; ^6^ LiSN- Listening in Spatialized Noise Test; Speech Reception Threshold (SRT) measured in decibels when Signal to Noise Ratio (SNR) results in 50% correct repetition of target sentence; ^7^ Absolute values shown for advantage measures; ^8^ Age-scaled scores used.
