# Correction: Hynes et al. “Reduced Educational Outcomes Persist into Adolescence Following Mild Iodine Deficiency in Utero, Despite Adequacy in Childhood: 15-Year Follow-Up of the Gestational Iodine Cohort Investigating Auditory Processing Speed and Working Memory” *Nutrients* 2017, *9* (12), 1354

**DOI:** 10.3390/nu11061272

**Published:** 2019-06-05

**Authors:** Kristen L. Hynes, Petr Otahal, John R. Burgess, Wendy H. Oddy, Ian Hay

**Affiliations:** 1 Menzies Institute for Medical Research, University of Tasmania, Private Bag 23, Hobart, TAS 7001, Australia; Petr.Otahal@utas.edu.au (P.O.); Wendy.Oddy@utas.edu.au (W.H.O.); 2 Department of Endocrinology, Royal Hobart Hospital, 48 Liverpool Street, Hobart, TAS 7000, Australia; J.Burgess@utas.edu.au; 3 School of Medicine, University of Tasmania, 17 Liverpool Street, Hobart, TAS 7000, Australia; 4 Faculty of Education, University of Tasmania, Locked Bag 1307, Launceston, TAS 7250, Australia; I.Hay@utas.edu.au

The authors wish to make a correction to the published version of their paper [1].

While responding to an editorial request to include additional labelling to Figure 1 in the final proof, one graph “(C) Reading” was accidently duplicated where graph “(D) Writing” should have been. A correct version of the figure is shown below.

The authors apologize to the readers for any inconvenience caused by the change. This change does not impact on the text of the paper or on the overall results or scientific conclusions. The original manuscript will remain online on the article webpage, with a reference to this correction.

## Figures and Tables

**Figure 1:**
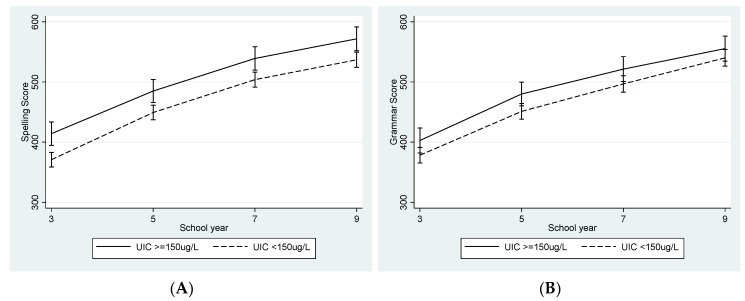

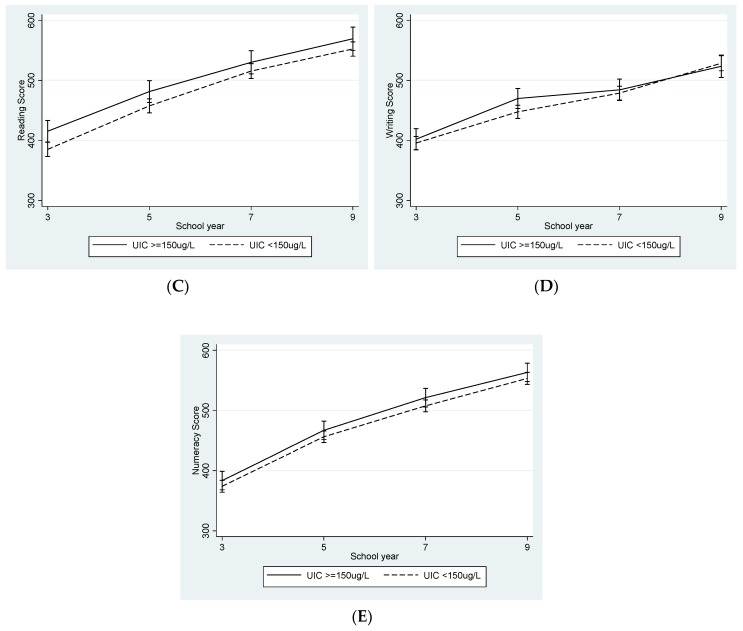
National Assessment Program – Literacy and Numeracy (NAPLAN) Scores for (**A**) Spelling, (**B**) Grammar, (**C**) Reading, (**D**) Writing, and (**E**) Numeracy from School Year 3 to Year 9 by maternal urinary iodine concentration (UIC), using data from the final mixed-effects multiple imputation using chained equations (MICE) models adjusted for biological factors, socio-economic status (SES), and adolescent UIC (*n* = 266).
